# Phenotypic Profile of Rh and Kell Blood Group Systems among Blood Donors in Cote d'Ivoire, West Africa

**DOI:** 10.1155/2014/309817

**Published:** 2014-09-24

**Authors:** L. Siransy Bogui, B. Dembele, Y. Sekongo, S. Abisse, S. Konaté, M. Sombo

**Affiliations:** ^1^National Blood Transfusion Center, 52 boulevard de Marseille, BP 15 Abidjan, Cote d'Ivoire; ^2^Laboratory of Immunology, UFR of Medical Sciences, BP 34 Abidjan 01, Cote d'Ivoire; ^3^Laboratory of Immunology, UFR of Pharmaceutical and Biological Sciences, BP 34 Abidjan 01, Cote d'Ivoire; ^4^Hospital and University Centre of Cocody, BP 1843 Abidjan 08, Cote d'Ivoire

## Abstract

Few countries in sub-Saharan Africa make systematic searches for antigens C, c, E, and e of the Rh and Kell system antigens in the donor and recipient, thereby exposing transfused patients. *Purpose and Objectives*. In this paper, we propose to determine the red cell Rh and Kell blood groups among blood donors from traditional techniques to improve medical care of transfused patients. This study will allow us to assess the frequency of blood group antigens in these systems. *Study Design and Methods*. We carried out a study on the red cell typing in the blood donor population of the National Blood Transfusion Center in Abidjan. This study was performed on 651 blood donors. *Results*. For the Rh system, the antigen frequencies of D, c, e, C, and E are, respectively, 92.93%, 99.85%, 99.85%, 21.97%, and 13.82%. K antigen is found in 0.77% of donors. *Discussion and Conclusion*. Although the frequencies of the most immunogenic antigens are lower than in the white race, lack of preventive measures makes the immunological risk high in Africa. Furthermore, Africa is full of specificities that are important to note for a better care of our patients.

## 1. Introduction

In Cote d'Ivoire and in other African countries [1, 2], most of transfusions are done only based on ABO and D antigens. Although blood transfusions can save life, they are not without risk. Blood transfusion can carry immediate or delayed immunological risks; the most common and most serious is the hemolytic transfusion reaction by antibody incompatibility. Knowledge about the frequency of red cells antigens phenotypes in Ivorian population is important for the creation of a donor data bank and to minimize risks of alloimmunization. This requires the determination of the immunological characteristics of blood products and blood recipients by performing immunohematology analysis such as phenotyping in Rh and Kell blood group systems. Currently, there are thirty-three major blood group systems [3], but analyses recommended in the usual situation are ABO, Rh, and Kell typing and detecting red cell antibodies.

Unfortunately, in sub-Saharan countries, few practice this systematic search for antigens C, c, e, E, and K in the donor and recipient, thereby exposing the transfused patient to high risk of alloimmunization [4].

Very few studies are available, reporting antigens frequencies of Rh and Kell blood groups in sub-Saharan countries.

This study is the first report on the frequency of blood groups system Rh and Kell in blood donors in Cote d'Ivoire. This work will perform Rh and Kell red cell typing among blood donors by traditional techniques to implement this into the routine for blood donors and recipients. It also allows us to determine the frequency of the major Rh and Kell blood group antigens and phenotypes commonly found among blood donors from Cote d'Ivoire to improve transfusion practices.

## 2. Materials and Methods

It was a retrospective study conducted at the Laboratory of Immunohaematology of the National Blood Transfusion Center of Abidjan, the capital of Cote d'Ivoire.

### 2.1. Blood Donors

We analyzed grouping data in Rh and Kell blood group systems from 651 volunteer regular blood donors coming to the national blood on one year. The blood donors have an age range from 18 to 60 years.

Those excluded from donating blood fell within the following categories:

- taking drugs for high blood pressure or heart failure;
- having Hb below 11 g/dL (for females) or 12 g/dL (for males);
- testing positive for HBsAg, HCV, and HIV antibodies and syphilis;
- having had jaundice, liver disease, epilepsy, diabetes, duodenal or gastric ulcer, asthma, tuberculosis, or other pathology;
- taking self-injected drugs;
- having sickle cell disease;
- being a prostitute and/or homosexual;
- having severe weight loss within the last six months.

### 2.2. Methods

For optimal results, the determination was performed using a tube freshly drawn into ethylenediaminetetraacetate according to manufacturer's instructions. The techniques are direct agglutination of the antigens with slide technique for Rh system antigens and indirect antiglobulin technique by tube technique for Kell system antigens. Rh phenotyping was done using five monoclonal monospecific antisera: anti-D, anti-E, anti-C, anti-c, and anti-e while Kell phenotyping was performed with anti-K according to manufacturer's instructions. All reagents were supplied by Orgenics PBS, Eurobio.

Positive and negative control red cells and Coombs' control cells were also performed as controls. Data were entered and analyzed with Epi Info version 6.1.

## 3. Results

We determined blood group antigens in 651 donors with sex ratio 3.6 in favor of men.

**Figure 1:**
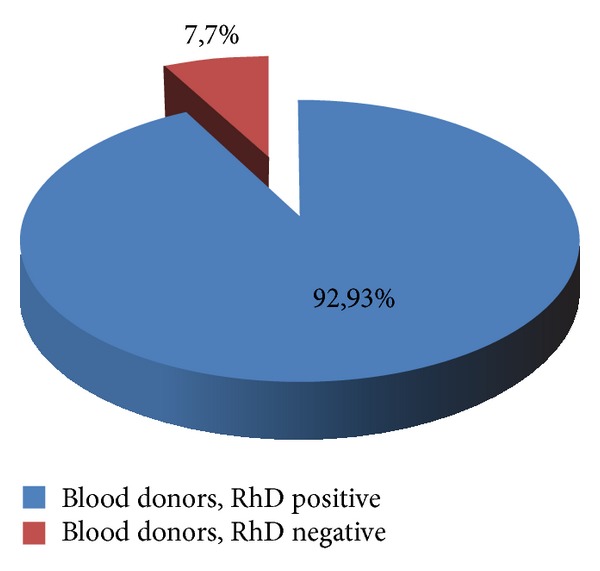
Prevalence of RhD antigen in blood donors.

605 blood donors representing 92.93% of the blood donors were found to be RhD positive while 46 blood donors representing 7.07 were found to be RhD negative (Figure 1).

The c and e antigens have the highest frequency with 99.83%. C and E antigen were less frequent with 21.97% and 13.82%, respectively (Table 1).

Seven phenotypes were detected among the blood donors (Table 2). The most frequent phenotype among the RhD positive was R_0_r 65.12% followed by R_1_r 20% and R_2_r 12.73%. Among the RhD negative, the most frequent was rr (80.43%).

In the Kell blood group system, 5 blood donors (0.77%) were typed as K antigen positive and 645 (98.08%) as k antigen positive antigens. Accordingly, the K−k+ phenotype was the most common in these donors (98.92%).

## 4. Discussion

Our study focused on 651 regular and volunteer blood donors who have made donations at the National Blood Transfusion Center. The techniques used were the traditional techniques of agglutination on slide or in tube (indirect antiglobulin). Although recent years have been marked by the appearance of microtechnology, we wanted to show that, even with traditional techniques, such typing can be performed in a department with limited resources. The findings in our study will introduce plan for better care of the patients.

### 4.1. The Rh System

After ABO blood group, the Rh system is the most important in transfusion medicine. In Cote d'Ivoire, the blood transfusions are done only regarding ABO and RhD antigens exposing patients to high alloimmunization. Akre [4] found that 62.8% of patients suffering from sickle cell disease and transfused were immunized against Rh and Kell antigens systems. Rh was involved in 44.44% while Kell was involved in 27.78% in sickle cell patients transfused against 38% for both in France [12]. The most frequent alloantibodies were anti-E, anti-C, and anti-KEL1 developed after transfusion of standard red cell units.

In our study, the frequency of D antigen was 92.93%. This is comparable with the findings in the north of Cote d'Ivoire [3] and in the black population [13]. It is higher in other sub-Saharan Africa countries [1, 2, 5] and non-sub-Saharan Africa countries [7–10] (Table 3). Frequencies are lower in the US, France, and Nigeria where the authors found respective prevalence of 85.4%, 85%, and 81.5% [6, 11, 12].

The prevalence of the RhD negative is 7.7%. These results agree with the work of Seka [14] who found 7.28% and the work of Cabannes [15, 16] that quoted values ranging from 1.70 to 9.3% in sub-Saharan Africa.

The frequencies of c and e antigens in our study are high (99, 85%) (Table 1) while the frequencies of C and E antigens are lower, respectively, 21.97% and 13.82%. Among whites, European, and Asian people, e antigen is the most popular, and then comes C antigen [17]. As regards to C and E antigens, frequencies are higher than in our study (C 70% and E 26%) [5, 9, 12, 17]. Among RhD negative donors, E antigen is absent.

In the Rh system, seven phenotypes on eighteen known [13] were identified in our population (Table 2). The phenotype most frequently encountered is the phenotype R_0_r (65.12%) regarding RhD positive blood donors and rr 80.43% among RhD negative blood donors. This profile is different from that observed in the whites where the phenotype R_1_r or R_1_R_1_ is the most popular [12, 13]. R_z_R_z_ was found in one donor.

### 4.2. The Kell System

The importance of this system is due to the K antigen, which has strong immunogenicity. It is among the most immunogenic system after the Rh system.

In the Kell system, k antigen was found in 98.92% of blood donors which is comparable with frequency in whites.

The prevalence of K antigen was found to be 0.92% of donors enrolled in the study, contrary to France where 9% of individuals are K+ [12] and to Germany where 4% express the K antigen [18].

The most common phenotype was K−k+ which is also common in white people. The antigens Kp^a^ and Kp^b^ were found, respectively, at frequencies of 0.61% and 82.80% in our sample, against 0.2% and 99.8% in whites [17].

## 5. Conclusion

Immunohematology data on blood donors are very few in West Africa. The ABO and D antigens are the main examination; other investigations are not performed.

Furthermore, Africa is full of its own specificities that are important to note for better care of patients by improving routine tests like phenotyping red cells, screening, and identifying red cells antibodies.

Research in that field should benefit from common work from West Africa national blood transfusion centers to enhance safety in transfusion medicine.

## Figures and Tables

**Table 1:** Rh and Kell antigens frequency among 651 Ivorian blood donors.

Antigens	RhD negative donors		RhD positive donors		Total	
	*n*	%	*n*	%	*n*	%
c+	46	100	604	99,83	650	99,85
e+	46	100	604	99,83	650	99,85
C+	9	19,57	134	22,15	143	21,97
E+	0	0	90	14,88	90	13,82

k					650	99,84
K					5	0,77
Kp^a^					4	0,61
Kp^b^					539	82,80

**Table 2:** Rh and Kell phenotypes frequencies in Cote d'Ivoire blood donors (*n* = 651).

Antigens	Phenotypes	Genotypes		Number	Frequency %
		Wiener	Fischer race		
Rh positive donors				605	
D+C−E−c+e+	R_0_r	R^0^r R^0^R^0^	Dce/dce Dce/Dce	394	65,12
D+C+E−c+e+	R_1_r	R^1^r R^1^R^0^ R^0^r′	DCe/Dce DCe/Dce Dce/dCe	121	20,00
D+C−E+c+e+	R_2_r	R_2_r R^2^R^0^	DcE/dce DcE/Dce	77	12,73
D+C+E+c+e+	R_1_R_2_	R^1^R^2^ R^1^r′′ R^2^r′ R^Z^r R^0^R^z^ R^0^r^y^	DCe/DcE DCe/dcE DcE/dCe DCE/dce Dce/DCE Dce/dCE	12	7,00
D+C+E+c−e−	R_z_R_z_	R^z^R^z^ R^z^r^y^	DCE/DCE DCE/dCE	1	0,17
Rh negative donors				46	
D−C−E−c+e+	rr	rr	dce/dce	37	80,43
D−C+E−c+e+	r′r	r′r	dCe/dce	9	19,57

Kell					
K−k+				645	99.08
K+k+				5	0.77
K−k−(k0)				1	0,15

**Table 3:** Antigens frequencies of Rh blood group compared with published results.

Antigen	Our study (*n*)	Our study (%)	North Cote d'Ivoire [3]	Mauritania [1]	Guinea [5]	Cameroun [2]	Nigeria [6]	Antananarivo [7]	Morocco [8]	India [9]	Iran [10]	USA [11]
D+	605	92,93	92.5	94,2	95,9	95	81,5	98,9	90,5	94,4	90,2	85.4
D−	46	7,70	7.5	5,77	4,06	6	18,5	1,1	9,5	5,64	9,8	14.6
c+	650	99,85				97,5					73,9	
e+	650	99,85				97,5					97,9	
C+	143	21,97				95					75,9	
E+	90	13,82				92,5					29,5	
